# Hair salons and stylist–client social relationships as facilitators of community-based contraceptive uptake in KwaZulu-Natal, South Africa: a qualitative analysis

**DOI:** 10.1186/s12978-021-01226-4

**Published:** 2021-08-30

**Authors:** Nafisa J. Wara, Christina Psaros, Sabina Govere, Nosipho Dladla, Ashley Stuckwisch, Dani Zionts, Jana Jarolimova, Ingrid V. Bassett

**Affiliations:** 1 Medical Practice Evaluation Center, Massachusetts General Hospital, 100 Cambridge Street, 16th Floor, Boston, MA USA; 2 Behavioral Medicine Program, Department of Psychiatry, Massachusetts General Hospital, Boston, USA; 3 Harvard Medical School, Boston, USA; 4 AIDS Healthcare Foundation, Durban, South Africa; 5 Division of Infectious Diseases, Massachusetts General Hospital, Boston, USA; 6 Center for AIDS Research (CFAR), Harvard University, Boston, USA

**Keywords:** South Africa, Contraception, Adolescent girls and young women, Hair salon, Community delivery

## Abstract

**Background:**

South Africa faces a high burden of unmet contraceptive need, particularly among adolescent girls and young women. Providing contraception in community-based venues may overcome barriers to contraceptive access. Our objective was to explore the potential impact of the social environment and stylist–client interactions on perceived accessibility of contraceptives within hair salons.

**Methods:**

We conducted 42 semi-structured, in-depth interviews with salon clients (100% identified as female, 100% identified as Black, median age 27.1 years) and 6 focus groups with 43 stylists (95% identified as female, 98% identified as Black, median age 29.6 years) in and around Umlazi Township, Durban, KwaZulu-Natal to explore perspectives on offering contraceptive services in hair salons. We used an inductive and deductive approach to generate the codebook, identified themes in the data, and then organized findings according to Rogers’ Individual Adoption Model as applied to community-based health prevention programs. Twenty-five percent of transcripts were coded by two independent coders to ensure reliability.

**Results:**

We identified elements of the salon environment and stylist–client relationships as facilitators of and barriers to acceptability of salon-based contraceptive care. Factors that may facilitate perceived contraceptive accessibility in salons include: the anonymous, young, female-centered nature of salons; high trust and kinship within stylist–client interactions; and mutual investment of time. Stylists may further help clients build comprehension about contraceptives through training. Stylists and clients believe salon-based contraceptive delivery may be more accessible due to contraceptive need facilitating client buy-in for the program, as well as a salon environment in which clients may encourage other clients by voluntarily sharing their own contraceptive decisions. The non-judgmental nature of stylist–client relationships can empower clients to make contraceptive decisions, and stylists seek to support clients’ continued use of contraceptives through various adherence and support strategies. Some stylists and clients identified existing social barriers (e.g. confidentiality concerns) and made recommendations to strengthen potential contraceptive delivery in salons.

**Conclusion:**

Stylists and clients were highly receptive to contraceptive delivery in salons and identified several social facilitators as well as barriers within this setting. Hair salons are community venues with a social environment that may uniquely mitigate barriers to contraceptive access in South Africa.

## Plain language summary

Women in South Africa experience high rates of unintended pregnancies. Because of high HIV prevalence within the country, women who experience unplanned pregnancies may also be vulnerable to HIV. Studies have detailed the barriers women in South Africa face in accessing contraceptives, including poverty, cultural norms around contraceptive use, lack of confidential spaces, and negative experiences with healthcare workers. In response, we propose the provision of contraceptive and preventive HIV care in salons—places that may be more convenient, accessible, and comfortable for women to acquire contraceptives. In this analysis, we aimed to understand how the salon social environment and client–stylist relationships may lower barriers women face when accessing contraceptives, using a framework for how individuals adopt new interventions. We conducted in-depth interviews with 42 salon clients and six focus group discussions with 43 stylists and used content analysis to explore themes within the data. Overall, participants identified components of the salon environment and stylist–client relationships that may facilitate how clients learn and make decisions about contraceptive use. These included perceptions of: salons being anonymous and centered around young, female clients; and stylists and clients recognizing contraceptive need within the community, being invested in salon services, and building trusted relationships. Although overall buy-in was high, some stylists and clients also identified barriers such as confidentiality concerns, negative views on stylists, and discomfort with discussing contraceptives. Overall, our study identifies hair salons as promising spaces for women in South Africa to seek contraceptive care.

## Background

From 2015 to 2019, an average of 121 million pregnancies per year, or 48% of all pregnancies globally, were unintended [1]. Women in sub-Saharan Africa are disproportionately impacted, with an annual rate of 91 unintended pregnancies per 1000 women aged 15–49 years, 30% higher than the global rate. Of these, nearly half occur among adolescent girls and young women (AGYW) ages 15–24 [2].

In South Africa, unintended pregnancy remains a major concern, with a high co-occurring burden of HIV and unintended pregnancy among women living with HIV [3–5]. Contributors to the high rate of unintended pregnancies among AGYW in South Africa include experiences of poverty, gender inequity, and gender-based violence impacting sexual health decision-making; and barriers to consistent use of and access to modern contraceptives, including negative perceptions of and experiences with healthcare providers [5–11]. Approximately one in five women aged 15–49 years in South Africa have an unmet need for contraception, highest among adolescent girls aged 15–19 at 31% and young women aged 20–24 at 28% [12].

Barriers to contraceptive use faced by AGYW exist interpersonally (e.g., in interactions with partners, peers, family, and community members) and structurally (within clinics and existing community-based contraceptive programs). Interpersonal barriers to contraceptive access in South Africa include cultural norms surrounding open discussion of contraceptive methods, conflicting messages regarding the effectiveness of condom use from authority figures, and pressure to conceive and/or obstruction of contraceptive use by male partners [6, 13–16]. While South Africa’s national contraception guidelines recommend youth-friendly services and mandate confidentiality [17], and despite contraceptives being available at no cost in public clinics, AGYW still face structural barriers to contraceptive access in clinic settings due to resistance, misinformation, and judgmental attitudes from healthcare workers and caregivers [6, 13, 16, 18–21]. Community-based settings may have the potential to improve contraceptive access; however, barriers can persist in such settings, including confidentiality (e.g. in school-based contraceptive programs) and insensitivity of community-based family planning programs to local contexts [6, 13, 20, 22–24].

Novel community-based venues for contraceptive delivery (i.e., on-site access to contraceptive services and/or promotion of contraceptive information) may have the potential to address interpersonal and structural barriers to contraceptive access and uptake. Hair salons in Umlazi, KwaZulu-Natal, South Africa have been previously identified as acceptable venues for delivery of contraceptives and HIV pre-exposure prophylaxis by salon owners, stylists, and clients due to their convenience and accessibility [2, 25]. We have previously assessed the feasibility and acceptability of providing health services in salons using cross-sectional descriptive data and qualitative analyses in the service of intervention design [2, 25]. While convenience and accessibility of salons may address structural barriers to contraceptive access, the social environment of salons may additionally impact contraceptive decision-making. Furthermore, while there are similarities between contraception and HIV prevention, there are also differences (e.g. availability and prior knowledge of prevention methods in community, stigma related to pregnancy versus HIV), leading to a need to assess the impact of salon-based delivery on contraception separately from HIV prevention. Thus, we were interested in the specific impact that hair salons as social spaces and interpersonal interactions within salons may have on the uptake of contraceptives. Using an analytical framework dedicated to assessing the behavioral process of decision-making in community-based prevention programs, we conducted a secondary qualitative analysis to understand how salons’ social environments and the interpersonal relationships between clients and salon stylists may uniquely impact decision-making around contraceptive uptake and access.

## Methods

### Study setting and participants

**Fig. 1:**
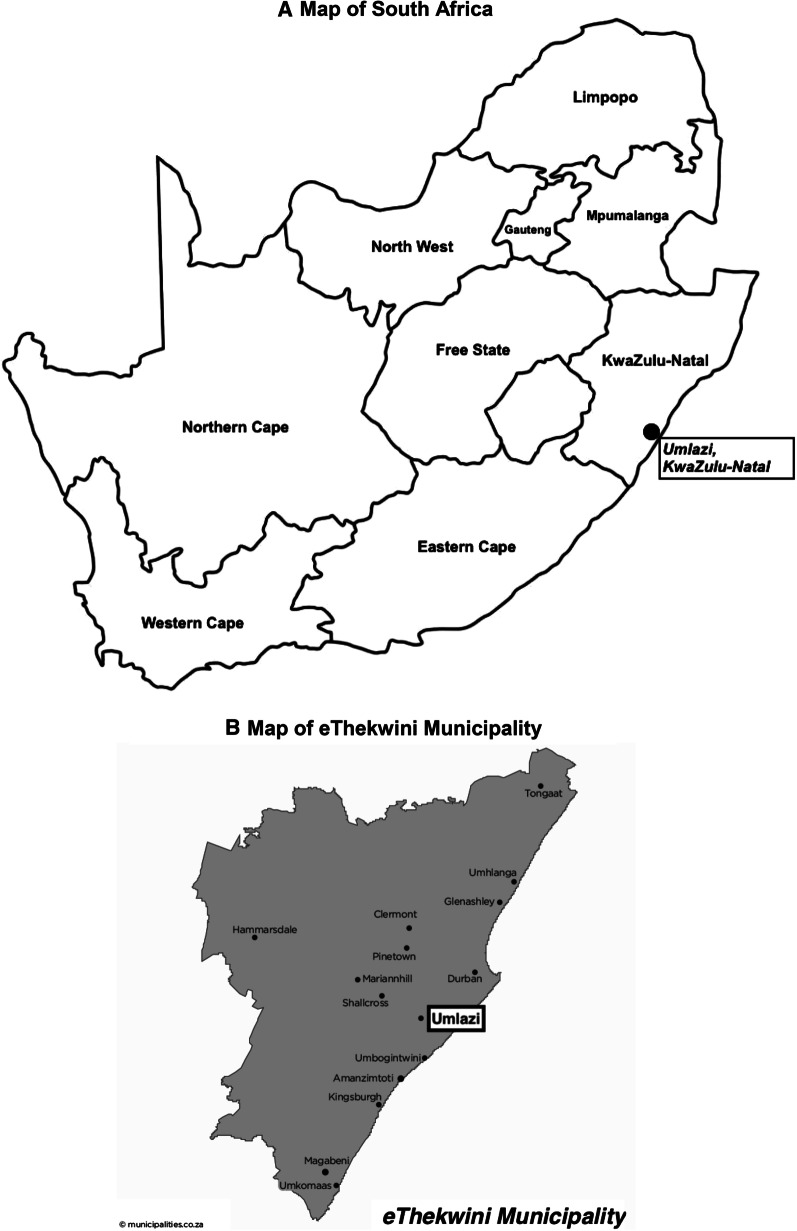
Geographical location of Umlazi (**A** generated by author, **B** source [27])

We conducted 42 individual, semi-structured, in-depth qualitative interviews with salon clients and six focus groups with 43 stylists in five hair salons in and around Umlazi, the second most-densely populated urban township in South Africa [26]. Located 20 km south of the city of Durban (Fig. 1) [27], Umlazi was assessed by the eThekwini Municipality in 2011 to have high rates of poverty (70% of households below the poverty line) and unemployment (above 50%) in Umlazi [28]. Umlazi had a population of > 400,000 per the 2011 census, with roughly 37,000 women ages 15–24 [29], although the estimated population has ranged as high as 2 million when accounting for individuals living in informal settlements within the township [30]. Hair salons in Umlazi vary in size and setup, including single- or multi-room locations within shopping malls, shipping containers, permanent or temporary buildings, and salons operating from within stylists’ homes. Within Umlazi, contraceptives and family planning services are available through free public- and paid private-sector clinics.

Interviews were conducted from September 2017 to May 2018. Data collection procedures were previously described in detail [2]. The interviewer (ND) was a female research assistant fluent in English and isiZulu and trained in qualitative interviewing. Participants were identified using purposive sampling methods from a convenience sample of hair salons identified by research staff while traveling through Umlazi and neighboring communities. Inclusion criteria for interviews and focus group participation required that participants be 18 years or older, speak English or isiZulu, and provide informed consent. The research assistant approached stylists and clients in salons to assess interest and obtain signed informed consent. Interviews and focus groups were audio-recorded, transcribed, and translated from isiZulu to English by an independent transcriber and reviewed by the South Africa-based study team (SG, ND) for quality of translation.

We developed semi-structured in-depth interview and focus group guides using guidelines by Huberman and Miles [31] for qualitative data collection, which were reviewed by the senior investigators and South Africa-based team members. The guides were piloted on six South Africa-based team members and evaluated on interview length, clarity of interview questions, and preparation for potential responses and questions from participants. The guides for in-depth interviews with salon clients included questions on contraceptive use and preferences, as well as opinions on offering contraceptive and other health services in hair salons. Stylist focus group guides included questions on their perceived role as hair stylists in the community, comfort with intervention participation, and resources for supporting the delivery of contraceptives and other health services in the salon. Example questions are found in Table 1.

**Table 1:** Sample study content areas and sample questions/probes for salon stylists and clients

Content area	Sample questions and probes
*Stylists*	
Warm-up questions	How do you think people perceive the role of the hair stylist in the Durban community?
	How do you perceive your role as a hair stylist?
	How would you describe the relationships you have with your clients?
Programmatic questions	Do you think discussion of health topics and offering services to clients at the salon is feasible?
	How do you think this would affect logistics and flow of clients through the salon?
	What kind of support might make you feel more comfortable? For example, having a health care provider on site to answer questions
Contraceptive care	What kinds of things can make it easy for women to get access to contraception? What kinds of things can make it hard?
	What resources might be useful to you as stylists for supporting offering contraception in the salon (i.e. scripts, promotional materials, posters, etc.)? How could these be implemented?
	What do you think about offering some sort of incentive or compensation for offering and accepting contraception at the salon?
	Do you think having adherence support (e.g. SMS reminders, support groups, incentives) for contraception would be helpful?
*Clients*	
Warm-up questions	What do you think are the major health care services that young women in Durban need?
	Of the services that you mentioned, are there any that you think you would be interested in receiving at the hair salon?
Contraceptive care	Could you describe your current contraceptive use (including your current contraceptive method, duration of use, how you chose your current method, your perceived need for contraception, and your interest in contraception)?
	What contraceptives are most attractive to you (oral contraceptive pills, injectables, hormonal subdermal implants, intrauterine devices)?
	Do you see hair salons as acceptable venues for contraception access and support? Why or why not?
	What are your preferences for who to hear reliable information about contraception from at the salon (i.e. hair stylist, peer mentor, nurse)? Why?
	Do you think having adherence support (e.g. SMS reminders, support groups, incentives) for your contraception would be helpful?

Study procedures were approved by the University of KwaZulu-Natal Biomedical Research Ethics Committee (BE388/16) and the Mass General Brigham (Massachusetts General Hospital/Brigham and Women’s Hospital) Institutional Review Board (Protocol 2016-P-001268, Boston, MA).

### Theoretical framework

As health communication about sexual and reproductive health is a primary component of contraceptive access, it is important to understand the ways in which the social environment of the salon would facilitate or impede an individual from gaining initial knowledge, forming an attitude, making a decision to accept or reject the service, and further confirming their decisions [29]. Rogers’ Individual Adoption Model was used to organize and interpret findings related to the impact of social dynamics present in salons and stylist–client interactions on the delivery of contraceptive information and contraceptive decision-making [32]. The Individual Adoption Model is a subcomponent of Rogers’ Diffusion of Innovation Theory, which explains how a group of individuals comprising a social system take up an innovation, leading to its diffusion through a community [32]. The Individual Adoption Model explains the process by which an individual responds to an innovation (e.g. an idea, behavior, invention perceived as new), from initial exposure to learning, leading to adoption or rejection of the innovation. This model has been used extensively in health communication research, as it highlights the importance of understanding how and why health information diffuses [32–35]. Further, we considered Green and McAllister’s (1984) adaptation of Rogers’ Individual Adoption Model to guide interpretation of the community structures that would be needed to support each phase in the individual adoption model prior to initiating implementation, as detailed in Table 2, columns A and B [36, 37]. This application of the model continues to inform the planning of community health behavior programs by identifying potential facilitators that may increase uptake of health prevention strategies.

**Table 2:** Features within a community supporting the phases of Rogers’ individual adoption process [33], and application within salon-based contraceptive uptake model

A	B	C
Phase in Individual Adoption Process (Rogers, 2002) [32]	Supporting Features of Community	Application within salon-based contraceptive uptake model
1. Exposure	Social setting with access to media	Salon as social environment with comfortable stylist-client, client-client interactions
2. Attention	Interest of family, peers, and other significant persons	Salon stylists as individuals trusted by clients introduce contraceptive uptake to clients
3. Comprehension	Group discussion and feedback, question and answer sessions	Stylists trained in health promotion discussing contraceptives one-on-one with clients, supplemented with educational materials (e.g. posters, graphics) to facilitate client knowledge
4. Belief	Direct persuasion and social influence, actions of informal leaders	Perception among women within community of the need for and benefits of contraceptives, high stylist buy-in to salon-based contraceptive delivery
5. Decision	Group decision making, public commitments, and repeated encouragement, which build self confidence	Clients may be encouraged by others who choose to disclose individual contraceptive decision-making, and not discouraged by stylists due to stylists’ nonjudgmental approach to contraceptive use by young women
6. Learning	Demonstrated and guided practice with feedback and continued confidence, advice, and direct assistance	Guided usage and assistance through SMS reminders to refill prescriptions, support groups among those taking contraceptives

Focusing on contraceptive delivery in hair salons as the innovation of interest, we sought to explore salon clients’ and stylists’ decision-making processes regarding contraceptives, in order to better understand the potential for diffusion and uptake of the innovation by other community members. We applied Green and McAllister’s adaptation of Rogers’ framework, evaluating our data within six major domains: (1) exposure to the prevention innovation and related information in a social setting with access to media; (2) attention to the innovation facilitated by family, peers, and significant individuals in the target audience’s life; (3) comprehension of the innovation facilitated by group discussion; (4) belief in the feasibility of the innovation facilitated by social influence and informal leadership; (5) decision facilitation through group decision-making and encouragement; and (6) an individual’s continued learning about an innovation through guided practice, advice, and direct assistance. We used these domains to organize the characteristics of stylist–client social interactions based on each phase in the individual adoption process. We also incorporated recommendations made by clients and stylists within relevant domains to further facilitate contraceptive delivery in salons.

### Analysis

We conducted directed content analysis through an iterative multi-step process [38]. One team member (NJW) with experience in qualitative research reviewed 48 transcripts, and developed an initial codebook, which was revised through review and multiple rounds of discussion with two senior investigators with qualitative expertise (IVB, CP) as well as with the South Africa-based team (SG). The updated codebook was further revised through review and discussion with another team member with qualitative training (AS) who reviewed the 48 transcripts separately. Using NVivo qualitative software (version released in March 2020), two team members (NJW, AS) jointly coded 12 transcripts (25%), meeting after coding every 3 transcripts to discuss discrepancies, refine the codebook (providing definitions, adding additional codes, and indicating example quotes), and maintain an audit trail. This process was reviewed after each set of coded transcripts by the senior researchers on the team (IVB, CP). After agreement was reached on the coding scheme, two team members (NJW and AS) independently coded the remaining transcripts and met weekly to discuss questions that arose while coding.

The initial thematic summary, which was deductively developed following the adapted Rogers’ Individual Adoption Model, was iteratively and inductively revised based on review and discussion of individual coded excerpts and analytic memos, leading to the clarification of existing themes and addition of new themes that arose during coding and were not based on the interview guide (e.g. “clients choosing to invest time in salon care,” “stylists motivated by additional business”). The summary of themes was shared with South Africa-based team members for feedback and to incorporate additional context, as well as with the rest of the study team.

## Results

Participant characteristics were previously described [2]. Briefly, 100% (n = 42) of clients and 93% (n = 40) of stylists identified as female, 100% (n = 42) and 98% (n = 42) as Black, and the median ages of each group were 27.1 and 29.6 years, respectively. Data from 42 client interviews and six focus groups with 43 stylists were organized into themes reflecting each of the six domains within the adapted Rogers’ Individual Adoption Model and presented in order. Table 2, column C revisits the Rogers’ Individual Adoption Model, supplementing each stage of the individual decision-making process with examples from our results. Representative quotes are organized by theme and provided in Table 3.

**Table 3:** Quotes from study participants organized by qualitative theme

Code	Sub-code	Quote
*Theme 1: Exposure to contraceptive information facilitated by salon environment*		
Salon characteristics	Facilitators	Confidential space: ""They would be happy talking to us because the salon is the only place that we meet; I do not know where my clients live so I cannot go to their neighbours and gossip about them." [Stylist]
		Female-oriented: “I don’t think there is anything that would make me feel uncomfortable because in this salon we are all women and we know these things.” [Client, 22 yo]
		Comfortable around other clients: "I think these services can be offered at the salon because at the salon even when you are waiting you are with people that you know so even the conversations that you are having they tend to make you not worry about the time that you spending at the salon." [Client, 25 yo]
	Barriers	Concerns regarding confidentiality: "Salons are public places. Imagine you are here to get your prevention pills from the salon and you bump into someone that you know, even your neighbour, everyone would know that you getting contraception from the salon." [Client, 24 yo]
Client characteristics	Facilitator	Investment of time: "As working women we hardly get time to go to the clinic but with the salon we don’t take chances. We make it a must that we go to the salon because as women we value our beauty through our hair styles. So for this reason I think it’s a good thing." [Client, 28 yo]
Recommendations		Ensuring confidentiality: "I think a mobile clinic is the solution because no one can tell what you are doing inside." [Client, 18 yo]
*Theme 2: Attention to contraceptive information facilitated by stylist–client relationships*		
Stylist characteristics	Facilitators	Prioritize happiness of clients through services: "The role of the hair stylist is to always make sure that women always look their best and to keep the hair healthy and their self-esteem up.” [Stylist]
		Prioritize happiness of clients through friendliness: "They see us as helpful people in their lives because anytime that they feel down they always come to the salon to do their hair and suddenly they feel okay." [Stylist]
		Learning about clients' lives: “Sometimes our clients just come to the salon just because they want to sit and chat with us.” [Stylist]
		Connecting over shared experiences: "Personally, I am a person that loves to talk. Sometimes a client comes to the salon to do her hair and I automatically start a conversation talking about my problems. As I talk about my problems, the client becomes comfortable enough to start talking about their problems. We end up giving each other advice. This relationship is not only limited to myself and my clients. I am like this to everyone, even with my colleagues." [Stylist]
		Stylist investment of time: "When the clients come to the salon, we become counsellors to them because some clients spend like 3–4 h at the salon. So when you are with a client for so many hours, even the problems or situations that they have in their lives you can talk to them and advise them." [Stylist]
		Referring to clients like kin: "My clients are my friends; I perceive them as my friends and my sisters." [Stylist]
		Prioritizing client trust: "When the clients come to the salon, we become counsellors to them because some clients spend like 3–4 h at the salon. So when you are with a client for so many hours, even the problems or situations that they have in their lives you can talk to them and advise them." [Stylist]
	Barriers	Unfamiliar to newer clients: "With new clients it can be tricky because some clients are cheeky especially when you haven’t established any relationship with them. But for those who know you, they will be comfortable.” [Stylist]
Client characteristics	Facilitators	Trust in stylist: "To be honest [my preference to hear reliable information about contraception] would be my stylist because I trust her. In the world today you need someone you can trust." [Client, 24 yo]
	Barriers	Discomfort discussing contraceptives: "We have to come up with a way to approach clients because some perceive this topic as very delicate." [Stylist]
Contraceptive access outside of salons	Barriers	Treatment by clinic staff: "A clinic is a scary place, nurses are rude and judgmental." [Client, 24 yo]
*Theme 3: Comprehension—salon facilitates environment within which clients and stylists can learn about contraceptives*		
Stylists' and clients' and past contraceptive usage	Barriers	Preconceptions about contraceptive usage: "Well I have heard stories that the loop is not guaranteed that you will not get pregnant—some women have fallen pregnant with the loop inside them." [Client, 27 yo]
Client characteristics	Barriers	Stigma against education level of stylists: "Some of the clients have very low regard for us with the idea that since we do hair, we are uneducated." [Stylist]
Stylist characteristics	Barriers	Stylists perceived as untrustworthy or rude: "We might talk and say all that we like but hair stylists are not known for their ability to keep secrets. Stylists are always talking…" [Stylist]
		Perceived as preoccupied or busy: "The stylist already has a job, to do hair. Sometimes you would come to the salon seeking advice and find that the stylist is busy." [Client, 31 yo]
	Facilitators	Willingness to be trained: "You must provide training for us so that we are knowledgeable in the topics that we will be discussing with our clients. It would be very unfortunate and embarrassing to start a conversation with our clients and when they ask questions about what we are discussing, we can no longer respond." [Stylist]
Recommendations		Audiovisual media: "I think we should have posters and condoms. Posters and condoms can show clients that there is a health initiative going on in this salon. And when they see the posters the clients will ask about the health services that are being offered." [Stylist]
		Healthcare provider on-site: "I think having a nurse on site would help because if we encounter problems or difficult questions we can always ask a nurse." [Stylist]
		Peer mentors: "I think it’s a peer mentor. I believe a nurse is only trained to administer injections. Whereas a peer mentor is also trained to approach people and build relationships and explain health service to people." [Client, 29 yo]
		Stylist support for client education: "I think there should be trainings and counselling that educates the people on the benefits of this programme, this way they can come willing because they will understand that this programme is aimed at helping them." [Stylist]
*Theme 4: Belief that accepting contraceptives in salons is feasible*		
Stylist characteristics	Facilitators	Overall buy-in: "As soon as the clients are aware of the services that are offered they will come. Even now, my clients are asking about you, they asking for your number and asking when you will be coming back to the salon." [Stylist]
		Perceived contraceptive need: "We talked about receiving health services such as contraceptives and testing for HIV, then the benefits that can arise is in the decline of unintended pregnancies among the youth because the youth is the biggest audience in the salon." [Stylist]
		Stylist interest in services and knowledge: "This programme will also be helpful to us as stylist in the salon because we will also get information that will help us grow because there is nothing more valuable than information." [Stylist]
		Motivated by additional business: "I think this will help retain our client because a client will know that I have a treatment that I am receiving from that salon or from that stylist, this helps because it helps keep clients coming to this particular salon." [Stylist]
	Barriers	Lack of education or health-related training: "I think we would have challenges with the injection because we have no training in administering injections. We can maybe give people pills and condoms." [Stylist]
Client characteristics	Facilitators	Overall buy-in: "I am comfortable with receiving contraceptives at the salon." [Client, 35 yo]
		Perceived contraceptive need: "I think women need help with preventing pregnancy. There are a number of women who have children that were not planned; hence they sometimes cannot afford to provide for these children." [Client, 24 yo]
		Destigmatizing sexual health: "I think this programme should start in schools so that more people can understand that using contraceptives is not a bad thing. In fact using contraceptives is a good thing because people are having sex anyways so having protection is the most reasonable thing to do." [Client, 29 yo]
Recommendations		Trained individuals administering contraceptives: "The salons are open Monday to Sunday, you can have someone or a nurse coming on a Tuesday to administer the injection to willing participants because we cannot have someone from the salon injecting us." [Client, 24 yo]
*Theme 5: Decision-making process facilitated by existing stylist–client social relationships, encouragement by other clients sharing contraceptive decision-making*		
Salon characteristics	Facilitators	Observation and reinforcement in salons: "I think some other clients are not sure what to do in life, so the more they come to the salon and see other clients, they start to assimilate behaviour and even style in terms of dress code and hairstyle that they want to do." [Stylist]
Stylist characteristics	Facilitators	Non-judgment: “We don’t judge people that they are coming to the salon for a specific thing” [Stylist]
		Relating to clients: "I would prefer a stylist because we have developed a relationship with the stylist and we talk with the stylists. We are general comfortable with ordinary people like us." [Client, 28]
		Validating client decisions: "Everyone coming to the salon will be here for the same reason, to do their hair and it will only be after we have shared our information that the client may decide anything else. Even those who come to the salon because they don’t want to go to the clinics will receive the same treatment as those who are here to do their hair." [Stylist]
Client characteristics	Facilitators	Observation and reinforcement with contraceptives: “"I can say I had adequate information on using the injection as a contraception method because most of my friends are using the injection as prevention." [Client, 22 yo]
*Theme 6: Continued learning and guided contraceptive usage in salons*		
Recommendations		Adherence strategies: "I think an SMS would work perfectly because everyone can receive an SMS, you don’t have to have data/bundles to receive an SMS." [Client, 46 yo]
		Support groups: "A [mobile app] group is better because people would be free to voice their opinions and help each other." [Client, 24 yo]
		Incentives: "I don’t want to lie, people like getting free things, so if you say you are giving out vouchers or money, the place will be crowded with people." [Client, 22 yo]
		SRH supplies: "So instead of bring us stuff just to make us happy, you can bring us feminine stuff that would also educate us." [Client, 25 yo]
		Other health services: "I think there should be a programme that provides testing facilities for women. These should be facilities where women can test for all disease and facilities that are close to women and where women can feel comfortable. These facilities should provide testing services for HIV, Pap smear, and should also provide contraceptives because people are reluctant on going to the clinic." [Client, 24 yo]

### Framework Theme 1 (Exposure): Exposure to contraceptive information in salons is facilitated by the salon setting and client priorities, despite confidentiality concerns

Across interviews and focus groups, stylists and clients discussed the anonymous, youth-facing, and female-oriented social setting facilitated by salon stylists, and discussed how these aspects may be conducive to learning about contraceptives in salons. Stylists and clients discussed how the relative anonymity of the stylist–client relationship, in which salon clients and employees do not know where clients live or interact with their neighbors, may contribute to greater client willingness to discuss sensitive health topics such as contraception. Stylists and clients discussed how the female-centeredness and younger age-range of clients in salons may contribute to clients feeling more comfortable speaking about contraceptives with each other and with stylists, as it is a topic perceived to be well-understood by young women. Stylists and clients reported how some female clients actively choose to invest time in visiting the salon over other venues such as clinics, due to highly prioritizing hair care and beauty, thus facilitating more convenient access to contraceptive information for clients in such a venue.

However, some stylists and clients also noted confidentiality concerns as barriers to offering contraceptives in salons. Some stylists and clients described concerns about stylists and clients all knowing each other, as well as clients gossiping about each other or discriminating against one another due to being seen as sexually active. Clients and stylists made suggestions to address these concerns, including using additional privacy structures when providing contraceptives in salons, such as a mobile van or tents, using nontransparent medication packaging, and maintaining confidential patient files.

### Framework Theme 2 (Attention): Client attention to contraceptive information can be facilitated by positive components of stylist–client interactions

Stylists discussed facilitating positive peer relationships with their clients through prioritizing client happiness, friendliness, building trust through conversation, and sharing common experiences, leading to a relationship with their clients similar to that of a friend or family member. Stylists noted how the extended duration of some salon services and the tendency of clients to return to the same stylist allows stylists and clients to speak often and at length. Stylists prioritize the happiness of their clients as an essential component of their professional role, through ensuring client satisfaction with both the beauty services and conversation. Strategies mentioned to build trust included being friendly and willing to listen and sharing their own experiences with their clients, leading to a relationship where they can discuss a variety of topics. Many stylists referenced these trusted relationships with their clients when speaking about the feasibility of introducing contraceptive information to them. From the client perspective, this trust was exhibited in their preference to hear information about contraceptives from their stylists instead of nurses. This may be further strengthened by experiences shared between stylists and clients, as some stylists’ experiences in seeking contraceptive care were similar to those reported by clients. These elements may contribute to stylists and clients developing relationships over time, reflected in how they refer to each other as being friends or like family members.

However, some stylists also discussed concerns with approaching certain clients because clients may feel discomfort while discussing contraceptives or sexual health topics, especially newer or younger clients. Stylists indicated concerns of potentially losing clients over the discomfort with being asked personal questions. Stylists discussed being able to tell immediately whether a client would be a good candidate for discussing contraceptive information, based on whether they are rude or friendly during their initial interactions.

### Framework Theme 3 (Comprehension): Stylist training on and demonstration of contraceptive knowledge can facilitate client comprehension of contraceptive information within salons, as well as influence client preconceptions of contraceptives and of stylists

Many clients mentioned preconceptions regarding contraceptives, specifically related to side effects (or lack thereof) and perceived efficacy. These preconceptions developed through interactions with other community members as well as clients’ own previous experiences with contraceptives and may serve as barriers to uptake of contraceptives. Furthermore, both clients and stylists discussed clients’ perceptions of stylists being uneducated, particularly about contraceptives. Some stylists felt stigmatized by clients who do not respect their choice of profession and assume that they are not knowledgeable, while clients expressed concern that stylists would provide inaccurate information because they lack a more comprehensive understanding of contraceptive issues or are too preoccupied or busy to provide accurate information. Some clients indicated that stylists are rude or not trustworthy, discussing fears that they may not maintain client confidentiality.

Although there are barriers regarding pre-existing contraceptive knowledge and negative client perceptions of stylists, stylists discussed overcoming these perceptions through the provision of stylist training in sexual health education as well as through using additional information sources to supplement their conversations with clients. Some suggested direct counseling of clients on the benefits of receiving contraceptive care, in order to de-stigmatize its receipt. Many stylists reflected that seeing posters and pamphlets may help clients learn about the health services within the salon. Other suggested strategies to facilitate comprehension of contraceptive information included using iPads at salons to present informational material, as well as advertisements on television and radio about contraceptive services being provided in salons. Some stylists and clients suggested that a healthcare provider be present to help answer questions and facilitate client comprehension about contraceptives. However, some clients also indicated a preference for speaking to peer mentors, who are as knowledgeable as healthcare providers but may be better at explaining and answering questions than providers, as well as potentially more able to maintain confidentiality than stylists.

### Framework Theme 4 (Belief): Stylists and clients demonstrate belief in the feasibility of providing contraceptives in salons

Stylists and clients reported overall positive opinions of providing contraceptive services in salons for several reasons: perceptions of community contraceptive need, stylist buy-in regarding services for themselves, and stylists’ perception of a potential increase in business due to contraceptive delivery in salons. Stylists and clients discussed the need for contraceptive services for young women in Durban due to perceived prevalence of unwanted pregnancies, serving as a unifying, motivating factor for stylists and clients to provide and seek contraceptives in a more accessible environment. Other motivating factors included reluctance to go to the clinic, the potential transmission of HIV to children through giving birth, and a lack of knowledge of various contraceptive options. Overall, stylists believed strongly in the potential effectiveness of contraceptive delivery in salons, both for themselves as well as clients, facilitating informal leadership and a positive social influence. Many clients reported a similarly high interest in accessing contraceptive services in salons. Furthermore, some clients described the importance of framing contraceptive usage positively, to reduce stigma, shame, and embarrassment around sexual activity. Some stylists also discussed being interested in providing contraceptive services at the salon because it may motivate clients to return to their salon, thus creating a mutually beneficial relationship for both clients and stylists.

While many stylists believed that their clients may benefit from accessing contraceptives in salons, stylists and clients also discussed concerns regarding client perceptions that stylists are not trained to administer contraceptives after providing contraceptive information, which may impact client confidence in the program. Stylists and clients discussed strategies to mitigate these concerns, including having a non-judgmental nurse or other clinically-trained individual present at salons to administer the contraceptives (particularly injectable contraceptives) after introduction by the stylist. Some clients believed that if stylists are properly trained, they could provide services as well.

### Framework Theme 5 (Decision): Contraceptive decision-making is facilitated by empathetic and nonjudgmental stylist–client interactions, as well as a salon social environment where clients can feel motivated by the behaviors of clients around them

Stylists facilitate an environment for non-stigmatized contraceptive decision-making by creating non-judgmental environments, exhibiting empathy, and validating their clients in making decisions. In addition, stylists and clients may choose to share their contraceptive decision-making with other clients, which may encourage individuals to accept contraception in their individual decision-making process. Stylists discussed the importance of not judging clients for the reasons they come to the salons or the conversations that they have as a part of their professional role. Additionally, stylists discussed the importance of an empathetic approach when talking with clients through listening and relating their own experiences, which motivates clients to come back regularly to the salon for beauty services and may have the same effect for contraceptive care. Stylists discussed the importance of validating client decisions, emphasizing that clients have the choice to use the information provided by stylists and make their own decisions.

These interactions differ from the social relationships reflected on by some clients and stylists with healthcare providers: both indicated that nurses were rude, busy, and judgmental (e.g. shouting at patients for missing appointments, which discourages further clinic visits). Clients additionally noted that they are reluctant to go to clinic because people will think they are there for HIV care. These dynamics lead to some stylists and clients feeling reluctant to seek contraceptive care at the clinic.

Importantly, stylists and clients noted that clients may feel motivated to seek salon services based on what clients around them are receiving, and thus may be encouraged to accept contraceptives when seeing publicly advertised availability of contraceptives in the salon, or when other clients choose to openly discuss their experiences accessing contraceptive services in the salon. This was further supported by how clients acquired contraceptive information in the past through seeing peers accept contraceptive services. Other methods may be used to facilitate the sharing of contraceptive decision-making while protecting the confidentiality of individual clients, such as the usage of support groups that people can opt into, or the presence of champions (public health advocates). Discussion among clients regarding their individual contraceptive decision-making, while personal, may be supported by the comfortable, female-centered salon environment perceived by salon stylists and clients (Framework Theme 1 above).

### Framework Theme 6 (Learning): Continued contraceptive learning and guided usage within salons can be facilitated through adherence and other support strategies

Stylists and clients recommended strategies to support the continued receipt and guided usage of contraceptives in salons by clients. Recommended adherence mechanisms included SMS reminders to encourage clients to return to the salon on-time to receive additional contraceptives, as SMS is private and more accessible than mobile apps. However, participants also discussed mobile app-based support groups as venues to continue sharing experiences and having discussions with other clients to continue learning about contraceptive usage. Stylists and clients offered mixed perspectives on providing monetary incentives or free items as adherence support, indicating that such incentives may motivate clients to come to the salon, while others maintained that clients should feel motivated to care for their health without an additional incentive.

Clients suggested the expansion of health education and services in salons beyond contraceptives. Suggestions included more education on HIV/AIDS, protection against sexually-transmitted infections (STIs) during sexual activity, and nutrition. Clients also suggested that hygiene products, such as menstrual cups and sanitary pads, be provided. Other recommendations included expanding health services to include general check-ups, STI prevention, HIV testing, cancer screening and education, and provision of vaccines.

## Discussion

This study explores the social dynamics present in salons and between stylists and clients that may contribute to the acceptance and uptake of contraceptives in hair salons in Umlazi, South Africa, using Rogers’ Individual Adoption Model as adapted by Green and McAllister. Overall, we identified a number of unique factors present in salons and in client-stylist interactions that facilitate a comfortable environment for salon clients to learn about, accept, and continue the use of contraceptives. Social facilitators specific to the salon setting included: confidentiality, centering on women’s needs, overall comfort around salon clients, and a space where clients can share, observe, and be motivated by other salon client behaviors. Facilitators within stylist–client interactions included: stylist and client investment of time in salon; stylists building trust with clients through prioritizing client satisfaction, learning about clients’ lives, and providing nonjudgmental advice; willingness to discuss contraceptives due to perceptions of contraceptive need and normalization of sexual health; and stylists being invested in salon-based contraceptive delivery for personal use and the potential for additional business. These facilitators were in contrast to participant experiences with accessing contraceptives in clinic environments, which were described by many participants as negative or stigmatizing. Although some participants identified social barriers to contraceptive delivery in salons, such as confidentiality concerns, client discomfort with the topic, and negative perceptions of stylists, participants also provided recommendations that may mitigate these barriers, including allocation of private space for contraceptive delivery within the salon, training stylists to deliver contraceptive information and care, stylist support by healthcare providers or peer mentors, audiovisual supplements, and the use of additional adherence support (e.g. support groups, SMS messages, incentives). Overall, these data support the unique facilitation of contraceptive decision-making in the salon setting, as well as the further diffusion of this innovation through the social networks of salon stylists and clients (by way of stylists continuing to discuss the contraceptive program with clients, or clients choosing to share their contraceptive acceptance and decision-making process with other clients).

The unique environment within salons as both community-based and relatively anonymous social spaces, prioritization of seeking salon services by some clients, and the salon environment centered around the needs of younger women were identified as facilitators for clients’ initial exposure to contraceptive information. The community-based setting of salons and confidentiality of stylist–client interactions may mitigate stigma related to seeking contraceptive care and being perceived as sexually active, especially for younger women. Other health delivery programs in South Africa have taken advantage of the relative anonymity associated with public multi-use spaces to facilitate care that may be stigmatized, such as differentiated antiretroviral therapy (ART) delivery programs that provide ART in community-based locations (e.g. supermarkets, churches) instead of clinics, reducing the likelihood of a patient being associated with seeking HIV care [39].

We identified a number of elements in stylists’ interactions with their clients that establish them as peer-like individuals of significance in clients’ lives, facilitating a relationship of trust that may enable stylists to introduce contraceptives to their clients in a way that feels comfortable to both individuals. As many stylists and clients reported having strong relationships where they already discuss sensitive information with each other, discussing contraceptives in this context may be more comfortable for clients than in clinics. Although studies exist assessing the cost-effectiveness of non-clinic-based contraceptive service delivery in South Africa [40], there is a lack of studies assessing the social facilitators and barriers that may impact the success of such a model. Despite this, an assessment of factors associated with contraceptive use in the Western Cape identified high self-esteem as a predictor of women using effective contraception. Thus, high self-esteem linked to stylist–client interactions may facilitate a similar increase in likelihood of contraceptive uptake within salons [41].

Stylists and clients overall had high buy-in to the idea of contraceptive delivery in salons due to a number of factors, including perceived need for contraceptives among young women in the community, client perceptions of the importance of destigmatizing contraceptive care, and stylist perceptions that having such a program would allow clients to observe others seeking contraceptive access and reinforce their own decisions, as well as providing additional business for the salon. Other studies, including a social network analysis conducted in Gombe, Nigeria, have demonstrated a similar positive impact that social networks can have on influencing health behaviors, specifically contraceptive uptake [42]. The non-judgmental nature of interactions between clients and stylists is notably different from the interactions stylists and clients described having with healthcare providers when seeking contraceptive care. Negative interactions with healthcare providers, as well as interactions in which healthcare providers made contraceptive decisions for clients, have been described as barriers to contraceptive access [6, 20, 43]. Thus, there is a need for avenues of contraceptive delivery within which young women are not shamed or judged for seeking contraceptive care.

Clients and stylists demonstrated an interest in expanding salon-based delivery to other health services beyond contraceptives, including HIV prevention, cervical cancer screening, nutrition education, and vaccine provision. Due to high rates of HIV incidence among women in KwaZulu-Natal, the provision of both preventive HIV and contraceptive services may address community needs in targeting both high HIV incidence as well as high rates of unplanned pregnancies [2]. Literature also indicates a high prevalence of curable sexually-transmitted infections (STIs) in KwaZulu-Natal, particularly among age groups with higher HIV incidence, leading to a potential benefit in providing point-of-care STI testing in salons [44]. Additionally, studies have identified fragmented and unequal access to cervical cancer screening with very limited follow-up in KwaZulu-Natal and other provinces in South Africa, which may further indicate a demonstrated need for greater access to women’s healthcare beyond HIV and family planning services [45].

Although clients and stylists reported social facilitators to accessing contraceptive care in salons, participants also identified barriers to accessing contraceptive care in this setting. Some participants reported confidentiality as a potential barrier to accessing contraceptives in salons. However, stylists and clients made recommendations to mitigate these concerns, such as additional privacy structures (e.g. tents, mobile vans). Stylists can also be trained to maintain confidentiality, a strategy already implemented when training peer educators in Sub-Saharan Africa to discuss sensitive health information related to pre-exposure prophylaxis and HIV with AGYW [43]. Some participants discussed specific clients feeling uncomfortable discussing these topics. This barrier could be mitigated by training stylists to maintain a non-directive approach when introducing contraceptives and discussing contraceptive services further only if the client is comfortable. These approaches are incorporated in peer education training materials related to providing AGYW with HIV information and access to services [46]. Other described barriers to developing comprehension of contraceptives include previously-held misconceptions about contraceptives (e.g. efficacy, side effects) as well as perceptions of stylists held by some clients (e.g. stylists are uneducated or cannot main confidentiality). These barriers could be mitigated by stylists building trust with their clients over time, demonstrating contraceptive knowledge during client interactions (facilitated by stylist interest in being trained to have conversations about contraceptives with their clients), and comprehension support through multimedia (e.g. posters, pamphlets, videos) and on-site healthcare providers.

Certain aspects of contraceptive care necessitate the presence of a healthcare provider within the salon setting (e.g. assessing a client’s risk for and addressing side effects when initiating contraceptive use, administering injectable contraceptives, combining contraceptive care with HIV prevention), as implemented previously in community-based programs in South Africa [20, 22–24], and supported by participants expressing need for clinical support. Due to previous negative experiences with healthcare providers, our results emphasize the importance of training any healthcare provider present in nonjudgmental, non-directive, and non-stigmatized approaches to providing contraceptives, particularly to younger women. Similar considerations would apply to expanding a salon-based approach to other forms of specialized care (e.g. STI testing and treatment, cervical cancer screening) suggested by study participants, which would also require clinical support within salons. While stylist roles would thus primarily involve health education/promotion, as described with prior salon-based health interventions in the United States [47, 48], some stylists expressed a willingness to be trained in contraceptive care, and it is worth exploring further the potential non-clinical roles that stylists could fill (e.g. health promotion, condom distribution, distribution of existing prescriptions written by healthcare provider).

These findings align overall with the facilitators and barriers to salon-based health service delivery identified by Bassett et al. (2019) [25]; however, beyond identifying additional social factors specifically impacting contraceptive uptake, this analysis links components of the salon social environment to one another by understanding how they may impact each stage of the contraceptive decision-making process (e.g. components of the salon environment that facilitate client attention to and interest in contraceptive uptake, recommendations from stylists and clients that may mitigate barriers to comprehension). This analysis thus identifies the unique potential of the salon social environment to be particularly effective in the diffusion of contraceptive uptake among community members.

This study should be considered in the context of its strengths and limitations. The focus on perceptions related to contraceptives allowed participants to directly compare their experiences with previously obtaining contraceptive care in non-salon settings, allowing for an in-depth understanding of how the difference in the delivery setting may impact the uptake of a service with which many women are already familiar. We sampled participants in both stylist and client roles from multiple salons in Umlazi, KwaZulu-Natal, allowing responses to be compared across participant types. Finally, the usage of the adapted Rogers’ Individual Adoption Model in evaluating this intervention provides a novel perspective on ways that community-based venues may be able to overcome social barriers related to an individual’s ability to receive, comprehend, and make decisions about their health, particularly regarding sensitive or stigmatized topics. This framework could be applied to understand the effectiveness of other existing or planned community health interventions in overcoming such barriers. However, some limitations persist: firstly, as interviews and focus groups were conducted in the urban setting of Umlazi, these results may not be generalizable to other contexts, for example, rural KwaZulu-Natal. Furthermore, women below the age of 18 are at high risk for unplanned pregnancies and may experience unique social facilitators or barriers to accessing contraceptives; however, they were not included. Finally, more specific questions regarding social influences were not incorporated into the interview and focus group guides; as a result, there may be additional social factors impacting contraceptive accessibility in salons beyond those discussed in this analysis.

## Conclusions

Overall, this study provides a detailed examination of the social facilitators and barriers to contraceptive uptake within community-based delivery of contraceptives, which complement factors identified in previous studies assessing feasibility and acceptability of community-based prevention of HIV and unplanned pregnancy. Stylists and clients recommended strategies to maintain a confidential, comfortable, and trusted environment to obtain contraceptive knowledge and services free of judgment. These factors may have a substantial impact on the uptake of contraceptive care in salons, a novel community-based setting, in comparison to existing venues for contraceptive delivery. The use of the adapted Rogers’ individual adoption model as a framework illustrates the particular strengths of this contraceptive delivery model in facilitating the individual’s contraceptive decision-making process and the potential for broader diffusion and contraceptive uptake by community members. Furthermore, this analysis provides rationale for use of this framework for assessments of other existing or future community-based programs for contraceptive delivery. The findings from this study support salon-based delivery of contraceptives as a promising and accessible approach to addressing the urgent contraceptive needs of adolescent girls and young women in South Africa.

## Data Availability

The datasets used and/or analysed during the current study are available from the corresponding author on reasonable request.

## Abbreviations

AGYW: Adolescent girls and young women
HIV: Human immunodeficiency virus
STIs: Sexually-transmitted infections
ART: Antiretroviral therapy
